# Supplementary material for: Variation in Clinical Application of Hyperthermic Intraperitoneal Chemotherapy: A Review
Source: Cancers (Basel). 2019 Jan 11;11(1):78. doi: 10.3390/cancers11010078 (PMC6357036; doi:10.3390/cancers11010078)
Supplement: Supplementary file 1 [file cancers-11-00078-s001.pdf]

Supplementary Materials: Variation in Clinical Application of Hyperthermic Intraperitoneal Chemotherapy: A Review

Roxan F. C. P. A. Helderman, Daan R. Löke, H. Petra Kok, Arlene L. Oei, Pieter J. Tanis, Nicolaas A. P. Klaas Franken and Johannes Crezee

Table S1. CRC origin.

Institute, Country	Drug(s) and dosage (mg/m²)	Carrier solution	Drug schedule	Volume (L)	Flow rate (mL/min)	Temp. (°C)	Duration (min)	Delivery method	Median OS (months)	1-year (%)	3-year (%)	5-year (%)	Mortality (%)	No of HIPEC procedures	Reference
The Netherlands Cancer Institute, The Netherlands	MMC (35) or OXA (460)	Dianeal	½ / ¼ - ¼	NR	NR	42	30–90	Open	37.2 (MMC) 29.4 (OXA)	NR	NR	NR	3.8	173	[1]
VU University Medical Center & Catharina Hospital Eindhoven, The Netherlands	MMC (35) or OXA (460)	NR	NR	NR	NR	37–41	30–90	Open or Semi-open	27	NR	NR	NR	1.1	345	[2]
15 Spanish HIPEC centers, Spain	MMC (30)	1.5% dextrose	NR	4	NR	42–43	60	Open, Closed, Semi-open	NR	NR	NR	NR	NR	NR	[3]
Mulicenter, Japan	CDDP (100) or MMC (10) and/or CDDP (50)	NR	NR	NR	NR	42	60	Open	NR	NR	NR	75.5	NR	42	[4]
The Netherlands Cancer Institute and Radboud University Medical Centre, The Netherlands	Until March 2014 MMC (35) After March 2014 OXA (460)	NR	NR	NR	NR	42–43	30-90	Open	51.7	NR	NR	NR	NR	414	[5]
Erasmus Medical Centre, Rotterdam, the Netherlands	OXA (260)	Dianeal	NR	2 L/m²	1000	42	30	Open or Closed	NR	NR	NR	NR	NR	NR	[6]
Severance Hospital & Gangnam Severance Hospital, Korea	MMC (35)	Dianeal: 1.5% dextrose	½ - ¼ - ¼	3	800-1000	42–43	90	Open	NR	NR	NR	NR	NR	66	[7]
National Cancer Centre Singapore, Singapore	MMC (10)	NR	NR	NR	NR	41–42	60	Closed	36	NR	NR	40.5	0	60	[8]
Manipal Comprehensive Cancer Center, India	5-FU (400) or OXA (300)	Saline and electrolyte free glucose	NR	NR	NR	42	30–90	Semi-open or Closed	76.9	NR	NR	NR	1.8	56	[9]
Saint-Luc University Hospital, Belgium	OXA (460) or MMC (35)	NR	NR	NR	NR	41–42	30–90	Closed	NR	89.5	NR	NR	0	52	[10]
Multicenter, The Netherlands	Before June 2014 MMC (35) After June 2014 OXA(460)	NR	NR	NR	NR	41	30-90	Open	NR	NR	NR	NR	0	43	[11]

University of California, San Diego, USA	MMC (10)	NR	NR	3-6	NR	42	90	Closed	NR	NR	NR	NR	NR	51	[12]
Lariboisière Hospital Paris, France	OXA (460) Recurrence: MMC	NR	NR	2 L/m^2	NR	42–43	NR	Closed	NR	NR	NR	NR	NR	341	[13]
St George Hospital in Sydney, Australia	OXA (350)	5% dextrose	NR	0.5	NR	42	30	Open	NR	NR	NR	26.8	2.3	184	[14]
National Cancer Centre Singapore, Singapore	MMC (35)	NR	NR	2-3	NR	42	60	Closed	27.1	83.7	38.7	19.1	NR	35	[15]
9 Dutch HIPEC centers, The Netherlands	OXA (460)	Isotonic dialysis fluid	NR	2	500	42–43	30	Open or Laparoscopic	NR	NR	NR	NR	NR	176	[16]
University of California, San Diego, USA	MMC (10 mg/L)	NR	$^2/_3$ - $^1/_3$	3–6	NR	42	90	Closed	NR	NR	NR	NR	1.4	70	[17]
Shiga University of Medical Science in Shiga, Japan	MMC (10 mg) + 5-FU (1000 mg)	Saline	NR	5	500-750	42–43	30	Open	NR	100	100	NR	0	5	[18]
Shiga University of Medical Science, Japan	MMC + 5-FU (90-130)	Saline	NR	5	500-750	42–43	30	Open	NR	66	NR	NR	0	9	[19]
Ghent University Hospital, Belgium	OXA (200-460) or MMC (35)	NR	NR	NR	NR	41	30-90	Open	27	NR	NR	NR	2.4	166	[20]
Milan National Cancer Institute & Bentivoglio General Hospital, Italy	CDDP (25ml/m^2/L^2)	NR	NR	4–6	700	42.5	60	Closed	NR	NR	NR	58.8	3	101	[21]
Wake Forest University Baptist Medical Center, USA	MMC (40 mg)	Crystalloid	$^3/_4$ – $^1/_4$	3	800-1000	40–42.5	120	Closed	43	NR	NR	NR	0	27	[22]
Zhongnan Hospital of Wuhan University, China	CDDP (20 µg/mL) + MMC (5 µg/mL)	Saline	NR	6	500	42.5–43.5	90	Open	16	70.5	22	22	0	60	[23]
Bentivoglio Hospital, Italy	CDDP (25) + MMC (3.3)	NR	NR	4–6	800-1200	42.5	60	Closed	NR	NR	NR	83.3	0	12	[24]
National Cancer Centre Singapore, Singapore	MMC	NR	NR	2-2.5	NR	42	60	Closed	NR	NR	NR	50.9	0	28	[25]
Aarhus University Hospital, Denmark	MMC (35)	Dialysis	NR	NR	NR	41–42.5	90	Open	NR	NR	47	38	1.3	34	[26]
The Valley Hospital Paramus, USA	MMC (30)	Dextrose	NR	NR	1500-2000	42	90	Closed	NR	NR	NR	NR	0	5	[27]
6 Belgian surgical centers, Belgium	OXA (460)	5% Glucose	NR	2 L/m^2	NR	41–42	30	Open	NR	97.5	NR	NR	0	48	[28]
Centre Hospitalo-Universitaire Lyon Sud, France	MMC (0.7 mg/kg) + IR	NR	NR	3–4	500	44–46 (Inflow)	90	Closed	NR	NR	NR	NR	0	12	[29]
5 Italian hospital and university centers, Italy	Between 1995-2005 CDDP (25) and/or	5% Dextrose	NR	2 L/m^2	NR	41.5–4 42–44	60-90 30	Open, Closed or Semi-open	21	NR	NR	NR	2.7	146	[30]

	MMC (3.3)<br>Between 2005-2007<br>OXA (460)														
St Agnes Hospital, USA & St George Hospital, Sydney, Australia	MMC (10-20)	NR	NR	NR	NR	42	90	Open	NR	85	48	NR	0	56	[31]
Cancer Center of Wuhan University, China	MMC (30)	Saline	NR	12	200	42.5–43.5	60-90	Open	NR	66.7	NR	30	0	21	[32]
University of Calgary, Canada	MMC (12-15 mg)	NR	NR	NR	NR	40–42	60	Open	NR	NR	36	NR	13	31	[33]
Poissy Medical Center, France	MMC (80 + CDDP (200)	0.9% NaCl	NR	3	NR	42–44	35-45	Laparoscopic	NR	86	NR	NR	0	21	[34]
Azienda Ospedaliera di Rilievo Nazionale ed di Alta Specializzazione Garibaldi, Italy	MMC (3.3) + CDDP (25)	NR	NR	4–5	600–1200	42.5	60	Closed	NR	NR	NR	NR	0	23	[35]
St Agnes Hospital, USA & St George Hospital, Australia	MMC (10-20)	1.5% Dextrose	NR	2–3	NR	42	90	Closed	13	NR	NR	NR	0	15	[36]
The Netherlands Cancer Institute, The Netherlands	MMC (35)	Isotonic	½ - ¼ - ¼	3	1000–2000	41-42	90	Open	NS	NS	NS	45	0	54	[37]
Uppsala University Hospital, Sweden	OXA (460)	50 mg/mL Glucose	NR	NR	NR	42-44	30	Open	NS	72.3	NR	NR	<1	38	[38]
Wake Forest University School of Medicine, USA	OXA (200-250)	NR	NR	NR	800–1000	40	120	Closed	NR	NR	NR	NR	NR	15	[39]
New Jersey Medical School, USA	DOX (15-100)	1.5% Dextrose	NR	4	1500–2000	40	90	Open	30.6	NR	NR	NR	0	9	[40]
University of Pittsburgh Medical Center, USA	MMC (40 mg)	Saline	⅔ – ⅓	3	NR	42	100	Closed	26.2	NR	49.9	NR	1.6	122	[41]
Institut Gustave Roussy, France	OXA	5% Dextrose	NR	2 L/m^2	1000	42-44	30	Open	60.1	NR	NR	NR	NR	NR	[42]
Department of Surgery of the University Regensburg, Germany	MMC (20) + DOX (15)	NR	NR	3	1000–1800	41-42	NR	Closed	NR	96	NR	NR	0	32	[43]
Giovanni Battista Antica Sede Hospital, Italy	MMC (15)	NR	NR	4-5	500–3000	42	60	Closed	30.3	64	NR	NR	4	25	[44]
St. George Hospital, Australia	MMC (10-12.5)	1.5% Dextrose	NR	2	NR	42	90	Open	29	72	NR	NR	0	30	[45]
Institut Gustave Roussy, France	OXA (460)	5% Dextrose	NR	NR	2000	43	30	Closed	60.1	NR	53	48.5	0	30	[46]
Institut Gustave Roussy, France	OXA (460)	5% Dextrose	NR	2 L/m^2	2000	42-44	30	Open	NS	74	NS	NS	0	24	[47]
Washington Hospital Center, USA	MMC (10-12.5) + 5-FU (650)	5% Dextrose	NR	2	NR	41-42	60	Closed	35	NR	NR	NR	0	34	[48]
The Netherlands Cancer Institute, The Netherlands	MMC (35)	NR	½ - ¼ - ¼	NR	NR	40-41	90	Open	21.8	75	28	19	6	117	[49]
Clinical Center of Serbia, Serbia	MMC (10-12.5)	Ringer's lactate	NR	2	NR	42	120	Open	15	NR	NR	NR	0	18	[50]
The Netherlands Cancer Institute, The Netherlands	MMC (35)	NR	½ - ¼ - ¼	3	1000–2000	41-42	90	Open	41.6	NR	NR	NR	2.9	102	(51]

Institut Gustave Roussy, France	OXA (460)	NR	NR	2 L/m²	2000	43	30	Open	NS	83	74	65	8	24	[52]
The Netherlands Cancer Institute, The Netherlands	MMC (35)	NR	½ - ¼ - ¼	3	1000–2000	41-42	90	Open	22.3	NR	NR	NR	8	105	[53]
University of Padova, Italy	MMC (3.3) + CDDP (25)	NR	NR	4-6	700–1000	41.5-42	90	Open or Closed	18	31	NR	NR	0	46	[54]
Centre Hospitalo-Universitaire Lyon Sud, France	MMC (0.7 mg/kg)	NR	NR	4-6	500	Inflow 46-48 Outflow 41-43	90	Closed	NR	79 (2-year)	NR	NR	1.8	26	[55]
China Medical University, China	5-FU (0.5 gr/L) + MMC (8 mg/L)	NR	¼ / 15 min	4	NR	43-45	60	Open	NR	NR	65.8	NR	0	46	[56]
Institut Gustave Roussy Comprehensive Cancer Center, France	OXA (260-460)	NR	NR	NR	2000	42-44	30	Open	NR	NR	NR	NR	0	22	[57]
The Netherlands Cancer Institute, The Netherlands	MMC (15-40)	Isotonic	NR	NR	1000	40-41	90	Open	NR	NR	23	NR	3	29	[58]
Université C.B. Lyon-I, France	MMC (10 mg/L)	NR	NR	4-6	400–500	Inflow 46-49 Outflow 41-43	90	Closed	12	NR	NR	NR	3.6	27	[59]
University General Hospital, Greece	CDDP (80-120)	NR	3 L/m²	4-9	NR	41-43	120	Closed	NR	NR	NR	NR	4	21	[60]
Centre Hospitalo-Universitaire Lyon Sud, France	MMC (0.7 mg/kg)	NR	NR	4-6	500	Inflow 46-48 Outflow 41-43	90	Closed	NR	NR	NR	NR	5.6	13	[61]

CDDP: Cisplatin, MMC: Mitomycin C, DOX: Doxorubicin, OXA: Oxaliplatin, 5: 5-fluoro-uracil, IR: Irinotecan , NR: Not Reported.

Table S2. Ovarian origin.

Institute, Country	Drug(s) and dosage (mg/m²)	Carrier solution	Drug schedule	Volume (L)	Flow rate (mL/min)	Temp. (°C)	Duration (min)	Delivery method	Median OS (months)	1-year (%)	3-year (%)	5-year (%)	Mortality (%)	No of HIPEC procedures	Reference
Danylo Halytsky Lviv NationalMedical University, Ukraine	CDDP (100) or CDDP (75) + DOX (15)	NR	NR	NR	NR	41.9–44.7	90	Closed	30.2	NR	NR	NR	6.8	59	[62]
Netherlands Cancer Institute, the Netherlands	CDDP (100)	NR	½ - ¼ / ¼	NR	1000	40	90	Open	45.7	NR	62	NR	0.8	122	[63]
Clinical Center of Serbia, Serbia	CDDP (15-30) + DOX (0.1-10)	Ringer's lactate	NR	2	NR	42	120	Closed	40.3	NR	65	24	0.8	60	[64]
Instituto de Medicina Integral Professor Fernando Figueira, Brazil	CDDP (25-240)	NR	NR	4-6	700	41–43	30	Closed	NR	NR	NR	NR	0	9	[65]
Zhongnan Hospital of Wuhan University, China	CDDP (100) + MMC (20) + PAC (100)	Saline	NR	6	NR	42.5–43.5	60	Open	57.5	97.8	65.2	56.5	0	46	[66]
University General Hospital, Spain	PAC (175)	NR	NR	NR	NR	42	60	Closed	NR	NR	NR	NR	4.76	21	[67]
Catholic University of the Sacred Heart of Rome & S. Orsola Hospital, Italy	OXA (460) or CDDP (75)	NR	NR	NR	NR	41.5	30–60	Closed	NR	NR	NR	52.8	0	70	[68]
University of Texas MD Anderson Cancer Center, USA	CDDP (100)	NR	NR	NR	NR	41	90	Closed	64	NR	NR	NR	0	101	[69]
7 French Cancer Centers, France	CDDP (50-80)	NR	NR	NR	NR	42	60	Open or Closed	27	NR	NR	NR	0	39	[70]
Institut Jules Bordet & Hôpital de la Citadelle, Belgium	CDDP (50)	NR	NR	NR	NR	42–43	60	Open	NR	92.3	NR	NR	0	16	[71]
AC Camargo Cancer Center, Brasil	MMC (10) + CDDP (50)	Dialysis	NR	4	NR	41–42	90	Closed	58.3	NR	NR	49.5	4	79	[72]
Cancer Hospital of Guangzhou Medical University, China	DOC (75) or CDDP (80)	Saline + 5% glucose	NR	3-7	450-600	41.5–42.5	90	NR	39	NR	NR	NR	0	53	[73]
University Hospital Bonn, Germany	CDDP	Saline	NR	3-4	1500	42–43	90	Open or Closed	NR	NR	NR	NR	0	NR	[74]
University Hospital Bonn, Germany	CDDP (60-100)	Saline	Single dose	3	NR	41–43	90	Closed	NR	67	NR	NR	0	12	[75]
Medical Park Samsun Hospital, Turkey	CDDP (75) or OXA (400)	NR	NR	3-5	NR	41–43	60–90	Closed	NR	95.8%	NR	NR	14.8	54	[76]
Metaxa Cancer Hospital, Greece	Platinum-sensitive CDDP (100) + PAC (175) Platinum-resistant	NR	NR	NR	NR	42.5	60	Open or Closed	26.7	NR	75	NR	0	60	[77]

	DOX (35) + PAC (175) or MCC (15)														
Gynecologic Oncology Associates, USA	CAR (6-10 AUC)	Saline	NR	NR	NR	41.5	90	Semi-open	5.8	NR	NR	NR	0	52	[78]
Catholic University of the Sacred Heart, Italy	OXA (460) or CDDP (75)	NR	NR	NR	NR	41.5	30-60	Closed	NR	NR	NR	NR	NR	22	[79]
Multicenter, Italy and Germany	CDDP (100) + PAC (175)	NR	NR	4–6	700	42.5	60-90	Open or Closed	32.9	NR	NR	NR	0	54	[80]
4 French Center, France	CDDP or ELO or MMC	NR	NR	NR	NR	42	30-60	NR	NR	NR	NR	75.6	0	23	[81]
Cancer Hospital of the Guangzhou Medical University, China	CCDP (50) + DOX (50)	Saline	NR	4.5–6	450–600	41-42	90	Closed	19	NR	NR	NR	0	17	[82]
Kantonsspital St. Gallen, Switzerland	CDDP (50)	NR	NR	NR	NR	42	90	NR	NR	NR	72.5	72.5	0	21	[83]
Didimotichon General Hospital, Greece	CDDP (50) + DOX (15) or GEM (1000)	NR	NR	3	2000	42.5-43	60-90	Open	37	NR	NR	54	0	43	[84]
National Cancer Institute Milan, Italy	Before 1997 CDDP (42 mg/L) + MMC (3.3) After 1997 CDDP (42 mg/L) + DOX (15 mg/L)	NR	NR	4-6	700	42.5	90	Closed	25.7	NR	NR	23	5.3	56	[85]
Ospedali Riuniti of Bergamo, Italy	CDDP (175) and/or PAC (175) and/or DOX (35)	NR	NR	NR	NR	41.5	90	Open	NR	95	60	NR	2.6	39	[86]
8 French centers, France	OXA (460)	Dextrose	NR	2 L/m^2	NR	42-44	30	Open	NR	93	NR	NR	0	31	[87]
Clinical Center of Serbia, Serbia	DOX (0.1 mg/kg) or CDDP (15)	Ringer’s lactate	NR	2	NR	40	120	Closed	38.1	NR	NR	67	3	56	[88]
National Cancer Center, Korea	CDDP (75)	1.5% Dextrose	NR	4	NR	41.5	90	Closed	NR	NR	NR	NR	0	30	[89]
Catholic University of the Sacred Heart, Italy	OXA (460)	5% Glucosate	Single dose	5	600-800	41.5	30	Closed	NR	96	NR	NR	0	25	[90]
Uppsala University Hospital, Sweden	CDDP (50) + DOX (15)	Dianeal	NR	NR	NR	42-44	30	Open	NR	72.3	NR	NR	0	6	[38]
University La Sapienza of Rome, Italy	CDDP (75)	NR	NR	4-6	500	42-43	60	Closed	30.4	NR	NR	16.7	4.2	45	[91]
Crete University Medical School, Greece	PAC (175)	Cremophor EL	NR	NR	NR	41-43	120	Closed	65.6	NR	NR	NR	0	13	[92]
Wake Forest University Baptist Medical Center, USA	CAR (400-1200)	Isotonic Saline	NR	2-3	600-800	39-43.5	90	Closed	NR	NR	NR	NR	0	17	[93]
National Cancer Institute of Milan, Italy	CDDP (43 mg/L) + DOX (15.25 mg/L)	NR	NR	NR	NR	42-43.5	60-90	Closed	NR	NR	NR	NR	0.9	41	[94]

4 Argentine gynecological services, Argentina	CDDP (100)	NR	NR	NR	400	41-43	60	Open or Closed	64.4	NR	NR	55	NR	29	[95]
San Giovanni Battista Antica Sede Hospital, Italy	CDDP (100-150)	NR	NR	4-6	900-1200	41.5-42.5	60	Closed	28.1	80	32	12	3.3	30	[96]
St. Mary's Hospital in the Catholic University of Korea, Korea	CAR (350)	Ringer's lactate	NR	6	NR	43-44	90	PCE	NR	NR	NR	63.4	0	117	[97]
National Cancer Institute of Milan, Italy	CDDP (43 mg/L) + DOX (15.25 mg/L)	NR	NR	NR	NR	42.5	60-90	Closed	NR	NR	NR	NR	0.6	36	[98]
BioMed-Hospital, Germany	CDDP 9100) or CAR (450)	Saline	NR	3-4	190-220	42-43	60	Closed	49	65	30	16	NR	36	[99]
National Cancer Institute, Milan, Italy	CDDP (25) + MMC (3.3)	Polysaline	NR	NR	600	42.5	60	Closed	NR	NR	71	NR	4	27	[100]
The Netherlands Cancer Institute, the Nehterlands	CDDP (50-70)	Saline	NR	3	1000	40	90	Open	NR	80	20	20	0	5	[101]

Table S3. Gastric origin.

Institute, Country	Drug(s) and dosage (mg/m²)	Carrier solution	Drug schedule	Volume (L)	Flow rate (mL/min)	Temp. (°C)	Duration (min)	Delivery method	Median OS (months)	1-year (%)	3-year (%)	5-year (%)	Mortality (%)	No of HIPEC procedures	Reference
Cancer Center of Guangzhou Medical University, China	5-FU (20 mg) + CDDP (100 mg)	Saline	NR	1-3	600	43	60	Open	NR	NR	NR	NR	NR	NR	[102]
Dutch Multicenter, The Netherlands	OXA (460) + DOC (0-150)	NR	NR	NR	NR	41-42	30	Open	NR	NR	NR	NR	NR	19	[103]
University Hospitals KU Leuven, Belgium	CDDP (100)	Saline	NR	3-4	NR	40-41	60	Open	16	71.9	14.1	3.5	0	32	[104]
Shiga University of Medical Science, Japan	5-FU (500-1000 mg) + MMC (10 mg) + CDDP (50 mg)	Saline	NR	5	1000	42	30	Open	NR	NR	NR	83.3	0	12	[105]
Hubei Cancer Clinical Study Center, China	LAB (50) + DOC (60)	Saline	NR	3	400	42.5-43.5	60	Open	14.3	58	40	NR	0	52	[106]
University Medical Center Regensburg or the St. John of God Hospital Regensburg, Germany	CDDP (75) + DOX (15)	NR	NR	NR	NR	42-43	60	Closed	17.2	71.1	24.1	6.4	0	38	[107]
Danylo Halytsky Lviv National Medical University, Ukraine	MMC (12.5) + CDDP (75)	NR	NR	NR	NR	41-43.6	90	Closed	NR	68.8	NR	NR	4.1	49	[108)
Multicenter, France	OXA (250)	NR	NR	2	NR	42-43	30	Open or Closed	NR	NR	NR	NR	NR	NR	[109]
Uppsala University Hospital, Sweden	CDDP (50) + DOX (15) or OXA (460)	Dianeal or 50 mg/ml Glucose	NR	NR	NR	42-44	90	Open	10.2	NR	NR	NR	0	18	[110]
Uppsala University Hospital, Sweden	CDDP (50) + DOX (15) or OXA (460)	Dianeal or 50 mg/ml Glucose	NR	NR	NR	42-44	90	Open	14.3	60	0	0	14	7	[111]
Zhongnan Hospital of Wuhan University, China	CDDP (60) + MMC (30)	Saline	NR	6	500	42.5-43.5	60-90	Open	11	41.2	14.7	NR	0	34	[112]
The Netherlands Cancer Institute, The Netherlands	MMC (35)	NR	NR	NR	NR	41	90	NR	NR	NR	NR	NR	NR	NR	[113]
Hubei Cancer Clinical Study Center, China	CDDP (120 mg) or MMC (30 mg) or HCPT (20 mg)	Saline	NR	12	200	42.5-43.5	90-120	Open	12	NR	NR	75	3.6	30	[114]
Ruijin Hospital Shanghai, China	CDDP (50 µg/ml) + MMC (5 µg/ml)	NR	NR	5-6	NR	42-44	60	Open	11.8	52.5	13.2	5.5	0	128	[115]
University Medical Center Regensburg, Germany	CDDP (75) + DOX (15)	NR	NR	NR	NR	42.5-43	60	Closed	NR	NR	NR	NR	0	11	[116]
Shanghai Jiaotong University, China	CDDP (50 µg/ml) + MMC (5 µg/ml)	NR	NR	5-6	NR	42-43	60	NR	60.85	83	70.5	67.9	0	118	[117]
National Cancer Institute of Milan, Italy	CDDP (25) + MMC (3.3)	NR	NR	NR	NR	42-43	60-90	Closed	NR	NR	NR	NR	0.9	12	[94]

Wake Forest University Baptist Medical Center, USA	MMC (40 mg)	Ringer's lactate	¾ - ¼	9	800-900	40-41	120	Closed	7.8	41	NR	17	0	34	[118]
Centre Hospitalier Lyon-Sud, France	MMC (40-60)	NR	NR	4-6	500	Inflow 46-48	90	Closed	10.3	48.1	NR	16	4	49	[119]
National Cancer Institute of Milan, Italy	CDDP + MMC or CDDP + DOX	NR	NR	NR	NR	42.5	60-90	Closed	NR	NR	NR	NR	0.6	12	[98]
Yokohama City University, Japan	CDDP (150) + MMC (15)	Saline	NR	5-6	NR	42-43	40	Open (PCE)	NR	NR	NR	49	0	124	[120]
Ludwig-Maximilians-University, Germany	CDDP (150) + MMC (15)	Ringer's lactate	NR	8	NR	43.5	60	NR	NR	NR	NR	NR	NR	24	[121]
Myongji Hospital, Korea	MMC (40 mg)	1.5% peritoneal dialysis solution	NR	4	400	42	120	Closed	NR	NR	NR	32.7	0	52	[122]
Washington Hospital Center, USA	MMC (10-25)	1.5% Dextrose	NR	3	1000	42-43	90	Closed	NR	89.2	NR	NR	1.5	7	[123]
Centre Hospitalo-Universitaire Lyon-Sud, France	MMC (10 mg/l)	NR	NR	NR	NR	Inflow 46-49	90	Closed	NR	80	41	NR	4.8	42	[124]
Social Insurance Funabashi Central Hospital, Japan	MMC (10 μg/ml)	Saline	NR	3-4	500-3000	43-45	120	Closed	NR	100	85	68	0	71	[125]
Tottori University, Japan	MMC (10 μg/ml)	NR	NR	NR	200	42-44	50-60	Closed	NR	95	65	60	0	42	[126]
Chiba Municipal Hospital, Japan	MMC (10 μg/ml)	NR	NR	3-5	NR	41-45	120	Closed	NR	80.4	24.5	NR	0	29	[127]
Tottori University School of Medicine, Japan	MMC (8-10 mg/l)	Salt solution	NR	8-12	100-200	40-44	50-60	Closed	NR	90	73.7	63	0	59	[128]

Table S4. MPM origin.

Institute, Country	Drug(s) and dosage (mg/m²)	Carrier solution	Drug schedule	Volume (L)	Flow rate (mL/min)	Temp. (°C)	Duration (min)	Delivery method	Median OS (months)	1-year (%)	3-year (%)	5-year (%)	Mortality (%)	No of HIPEC procedures	Reference
University of Maryland School of Medicine, USA	CAR (600) or MMC (40)	NR	NR	4-6	1500	41	90	Closed	32.8	70	49	36	2	100	[129]
French multicenter, France	CDDP (25) + MMC (3.3) or DOX (15) or OXA (460) + IR (360)	NR	NR	NR	NR	42-43	30-90	Open or Closed	61	NR	NR	53	5	24	[130]
St George Hospital, Australia	CDDP (100) + MMC (12.5)	Dianeal	NR	2	NR	NR	90	Open	NR	NR	NR	57.1	5.2	44	[131]
Scientific Institute for Cancer Research and Treatment, Italy	CDDP + MMC	NR	NR	NR	NR	NR	NR	NR	65	63	NR	44	7.1	42	[132]
Centre Hospitalier Lyon Sud, France	CDDP (20) + DOX (15)	NR	NR	NR	NR	42	90	Closed	NR	100	NR	NR	0	13	[133]
Centre Hospitalier Lyon Sud, France	CDDP + MMC	NR	NR	NR	NR	42-42.5	90	Closed	NR	NR	NR	NR	0	3	[134]
Fondazione IRCCS Istituto Nazionale dei Tumori di Milano, Italy	CDDP (25) + MMC (3.3) + DOX (15)	NR	NR	NR	NR	42-43	NR	Closed	NR	NR	NR	49	NR	116	[135]
St George Hospital, Australia	CDDP (50) + DOX (12)	NR	NR	NR	NR	42	90	Open	46	82	56	47	2	35	[136]
Wake Forest University Baptist Medical Center, USA	CDDP (250) or MMC (30-40 mg)	NR	NR	NR	1000	Outflow 40	60-120	Closed	40.8	NR	56	17	0	34	[137]
2 Italian referral centers, Italy	CDDP (25) + MMC (3.3) or MMC (35)	NR	NR	3.4-6	600-1000	40-42.5	60-90	Open or Closed	NR	NR	NR	100	0	12	[138]
National Cancer Institute Milan, Italy	CDDP (25) + MMC (3.3)	NR	NR	4-6	700	42.5	60-90	Closed	8	50	NR	NR	8	38	[139]
Uppsala University Hospital, Sweden	CDDP (50) + DOX (15)	Dianeal	NR	NR	NR	42-44	90	Open	NR	72.5	NR	NR	<1	5	[38]
Institut Gustave-Roussy, France	OXA + IR or CDDP	NR	NR	NR	1000	42-45	30	Open or Closed	100	NR	NR	63	4	26	[140]
National Cancer Institute Milan, Italy	CDDP (43 mg/L) + DOX (15 mg/L)	NR	NR	4-6	700	42.5	60	Closed	NR	100	90	90	0	13	[138]
National Cancer Institute Milan, Italy	CDDP (43 mg/L) + DOX (15 mg/L)	NR	NR	NR	NR	42-43	60-90	Closed	NR	NR	NR	NR	0.9	50	[94]
National Cancer Institute Milan, Italy	CDDP (25) + MMC (3.3)	NR	NR	3.5 L/m²	600	42.5	600	Closed	NR	88	72	55	0	49	[141]
National Cancer Institute Milan, Italy	CDDP (43 mg/L) + DOX (15 mg/L)	NR	NR	NR	NR	42-43	60-90	Closed	NR	NR	NR	NR	0	34	[98]
Centre Hospitalo-Universitaire Lyon Sud, France	MMC (0.5 mg/kg) + CDDP (0.7 mg/kg)	NR	NR	4-6	500	Inflow 44-49 Outflow 41-43	90	Closed	32	NR	NR	NR	0	5	[55]
National Cancer Institute Milan, Italy	CDDP (25) + MMC (3.3) or CDDP (25) + DOX (7)	NR	NR	4-6	600	42.5	60-90	Closed	NR	NR	69	NR	0	19	[142]

Table S5. PMP origin.

Institute, Country	Drug(s) and dosage (mg/m²)	Carrier solution	Drug schedule	Volume (L)	Flow rate (mL/min)	Temp. (°C)	Duration (min)	Delivery method	Median OS (months)	1-year (%)	3-year (%)	5-year (%)	Mortality (%)	No of HIPEC procedures	Reference
St George Hospital, Australia	MMC (10-12.5) or OXA (460)	1.5% Dextrose	NR	3	NR	42	90	Open	104	NR	NR	64	1.6	386	[143]
Fondazione IRCCS Istituto Nazionale Tumori, Italy	CDDP (25) or MMC (3.3)	NR	NR	4-6	700	42.5	60	Closed	NR	95	80	72	4	225	[144]
Princess Alexandra Hospital, Australia	MMC (35)	NR	NR	NR	NR	39.5	90	Open	NR	90	NR	NR	0	30	[145]
Fondazione IRCCS Istituto Nazionale Tumori Milano, Italy	CDDP (25) + MMC (3.3)	NR	NR	NR	NR	42-43	NR	Closed	NR	NR	NR	75	4.9	226	[146]
Basingstoke and North Hampshire Hospitals, UK	MMC (10)	NR	NR	NR	NR	42	60	Open	NR	NR	79.2	60.8	NR	519	[147]
Centre Hospitalier Lyon Sud Pierre Bénite, France	OXA (360)	NR	NR	NR	NR	42	30	Closed	NR	NR	NR	NR	0	36	[133]
Hospital University Reina Sofia, Spain	MMC (10-12.5)	1.5% Dextrose	NR	4	1000	41-43	60	Open	36	NR	NR	58.7	0	38	[148]
St George Hospital, Australia	CDDP (50) + DOX (12)	NR	NR	NR	NR	42	90	Open	46	82	56	47	2	118	[136]
Uppsala University Hospital, Sweden	MMC (10-35)	Dianeal	NR	Nr	NR	42-44	90	Open	NR	95	NR	NR	<1	47	[38]
Gustave Roussy Institute, France	MMC or OXA (460)	5% Dextrose	NR	2 L/m^2	NR	41-43	30-60	Open	NR	92	901	80	7.6	105	[149]
National Cancer Institute Milan, Italy	CDDP (25) + MMC (3.3)	NR	NR	4-6	700	42.5	60	Closed	NR	100	90	78.4	0	62	[150]
National Cancer Institute Milan, Italy	CDDP (25) + MMC (3.3)	NR	NR	NR	NR	42-43	60-90	Closed	NR	NR	NR	NR	0.9	49	[94]
Centre Hospitalier de l'Université de Montréal, Canada	MMC (0.5 mg/kg) + CDDP (0.7 mg/kg)	NR	NR	4-6	500	42-42.5	90	Closed	NR	100	78	52	0	27	[151]
National Cancer Institute of Milan, Italy	CDDP (25) + MMC (3.3)	Polysaline	NR	4-6	600	42.5	60	Closed	NR	97	97	97	3	33	[152]
National Cancer Institute of Milan, Italy	CDDP + MMC or CDDP + DOX	NR	NR	NR	NR	42.5	60-90	Closed	NR	NR	NR	NR	0	36	[98]
Istituto Nazionale per lo studio e la cura dei tumori, Italy	CDDP (25) + MMC (3.3)	NR	NR	NR	NR	42.5	60	Closed	NR	NR	NR	97	3	33	[153]
National Cancer Institute of Milan, Italy	CDDP (25) + MMC (3.3)	Polysaline	NR	NR	600	42.5	60	Closed	NR	NR	NR	NR	4.5	22	[154]
Wake Forest University, USA	MMC (30 mg)	Ringer's lactate	NR	2-3	NR	40.5	120	Closed	13.4	NR	NR	NR	NR	3	[155]

References:

1. van Eden, W.J.; Kok, N.F.M.; Woensdregt, K.; Huitema, A.D.R.; Boot, H.; Aalbers, A.G.J. Safety of intraperitoneal Mitomycin C versus intraperitoneal oxaliplatin in patients with peritoneal carcinomatosis of colorectal cancer undergoing cytoreductive surgery and HIPEC. *Eur. J Surg Oncol* **2018**, *44*, 220–227, doi:10.1016/j.ejso.2017.10.216.
2. Sluiter, N.R.; Rovers, K.P.; Salhi, Y.; Vlek, S.L.; Coupe, V.M.H.; Verheul, H.M.W.; Kazemier, G.; de Hingh, I.; Tuynman, J.B. Metachronous Peritoneal Metastases After Adjuvant Chemotherapy are Associated with Poor Outcome After Cytoreduction and HIPEC. *Ann Surg Oncol* **2018**, *25*, 2347–2356, doi:10.1245/s10434-018-6539-x.
3. Arjona-Sanchez, A.; Barrios, P.; Boldo-Roda, E.; Camps, B.; Carrasco-Campos, J.; Concepcion Martin, V.; Garcia-Fadrique, A.; Gutierrez-Calvo, A.; Morales, R.; Ortega-Perez, G., et al. HIPECT4: multicentre, randomized clinical trial to evaluate safety and efficacy of Hyperthermic intra-peritoneal chemotherapy (HIPEC) with Mitomycin C used during surgery for treatment of locally advanced colorectal carcinoma. *BMC Cancer* **2018**, *18*, 183, doi:10.1186/s12885-018-4096-0.
4. Kitai, T.; Yamanaka, K. Repeat cytoreduction and hyperthermic intraperitoneal chemotherapy for recurrent peritoneal carcinomatosis of appendiceal origin. *Int J Clin Oncol* **2018**, *23*, 298–304, doi:10.1007/s10147-017-1217-8.
5. Eden, M.W.J.v.; Elekonawo, M.F.M.K.; Starremans, B.B.J.; Kok, M.N.F.M.; PhD; Bremers, M.A.J.A.; PhD; Wilt, M.J.H.W.d.; PhD; Aalbers, M.A.G.J. Treatment of Isolated Peritoneal Recurrences in Patients with Colorectal Peritoneal Metastases Previously Treated with Cytoreductive Surgery and Hyperthermic Intraperitoneal Chemotherapy. *Annals of Surgical Oncology 25*, 1992–2001, doi:10.1245/s10434-018-6423-8.
6. Mehta, A.M.; Huitema, A.D.; Burger, J.W.; Brandt-Kerkhof, A.R.; van den Heuvel, S.F.; Verwaal, V.J. Standard Clinical Protocol for Bidirectional Hyperthermic Intraperitoneal Chemotherapy (HIPEC): Systemic Leucovorin, 5-Fluorouracil, and Heated Intraperitoneal Oxaliplatin in a Chloride-Containing Carrier Solution. *Ann Surg Oncol* **2017**, *24*, 990–997, doi:10.1245/s10434-016-5665-6.
7. Park, E.J.; Baik, S.H.; Hur, H.; Min, B.S.; Kang, J.; Han, Y.D.; Cho, M.S.; Lee, K.Y.; Kim, N.K. Cytoreductive surgery with hyperthermic intraperitoneal chemotherapy for appendiceal and colorectal cancer with peritoneal carcinomatosis: Clinical outcomes at 2 tertiary referral centers in Korea. *Medicine (Baltimore)* **2017**, *96*, e6632, doi:10.1097/MD.0000000000006632.
8. Bong, T.S.H.; Tan, G.H.C.; Chia, C.; Soo, K.C.; Teo, M.C.C. Preoperative platelet-lymphocyte ratio is an independent prognostic marker and superior to carcinoembryonic antigen in colorectal peritoneal carcinomatosis patients undergoing cytoreductive surgery and hyperthermic intraperitoneal chemotherapy. *Int J Clin Oncol* **2017**, *22*, 511–518, doi:10.1007/s10147-017-1092-3.
9. Somashekhar, S.P.; Prasanna, G.; Jaka, R.; Rauthan, A.; Murthy, H.S.; Karanth, S. Hyperthermic intraperitoneal chemotherapy for peritoneal surface malignancies: A single institution Indian experience. *The National medical journal of India* **2016**, *29*, 262–266.
10. Navez, J.; Remue, C.; Leonard, D.; Bachmann, R.; Kartheuser, A.; Hubert, C.; Coubeau, L.; Komuta, M.; Van den Eynde, M.; Zech, F., et al. Surgical Treatment of Colorectal Cancer with Peritoneal and Liver Metastases Using Combined Liver and Cytoreductive Surgery and Hyperthermic Intraperitoneal Chemotherapy: Report from a Single-Centre Experience. *Ann Surg Oncol* **2016**, *23*, 666–673, doi:10.1245/s10434-016-5543-2.
11. Simkens, G.A.; Verwaal, V.J.; Lemmens, V.E.; Rutten, H.J.; de Hingh, I.H. Short-term outcome in patients treated with cytoreduction and HIPEC compared to conventional colon cancer surgery. *Medicine (Baltimore)* **2016**, *95*, e5111, doi:10.1097/md.0000000000005111.
12. Baumgartner, J.M.; Kwong, T.G.; Ma, G.L.; Messer, K.; Kelly, K.J.; Lowy, A.M. A Novel Tool for Predicting Major Complications After Cytoreductive Surgery with Hyperthermic Intraperitoneal Chemotherapy. *Ann Surg Oncol* **2016**, *23*, 1609–1617, doi:10.1245/s10434-015-5012-3.
13. Voron, T.; Eveno, C.; Jouvin, I.; Beaugerie, A.; Lo Dico, R.; Dagois, S.; Soyer, P.; Pocard, M. Cytoreductive surgery with a hyperthermic intraperitoneal chemotherapy program: Safe after 40 cases, but only controlled after 140 cases. *Eur J Surg Oncol* **2015**, *41*, 1671–1677, doi:10.1016/j.ejso.2015.09.005.

14. Huang, Y.; Alzahrani, N.A.; Alzahrani, S.E.; Zhao, J.; Liauw, W.; Morris, D.L. Cytoreductive surgery and perioperative intraperitoneal chemotherapy for peritoneal carcinomatosis in the elderly. *World J Surg Oncol* **2015**, *13*, 262, doi:10.1186/s12957-015-0682-7.
15. Teo, M.C.; Ching Tan, G.H.; Lim, C.; Chia, C.S.; Tham, C.K.; Soo, K.C. Colorectal peritoneal carcinomatosis treated with cytoreductive surgery and hyperthermic intraperitoneal chemotherapy: the experience of a tertiary Asian center. *Asian J Surg* **2015**, *38*, 65–73, doi:10.1016/j.asjsur.2014.05.001.
16. Klaver, C.E.; Musters, G.D.; Bemelman, W.A.; Punt, C.J.; Verwaal, V.J.; Dijkgraaf, M.G.; Aalbers, A.G.; van der Bilt, J.D.; Boerma, D.; Bremers, A.J., et al. Adjuvant hyperthermic intraperitoneal chemotherapy (HIPEC) in patients with colon cancer at high risk of peritoneal carcinomatosis; the COLOPEC randomized multicentre trial. *BMC Cancer* **2015**, *15*, 428, doi:10.1186/s12885-015-1430-7.
17. Baumgartner, J.M.; Tobin, L.; Heavey, S.F.; Kelly, K.J.; Roeland, E.J.; Lowy, A.M. Predictors of progression in high-grade appendiceal or colorectal peritoneal carcinomatosis after cytoreductive surgery and hyperthermic intraperitoneal chemotherapy. *Ann Surg Oncol* **2015**, *22*, 1716–1721, doi:10.1245/s10434-014-3985-y.
18. Shimizu, T.; Sonoda, H.; Murata, S.; Takebayashi, K.; Ohta, H.; Miyake, T.; Mekata, E.; Shiomi, H.; Naka, S.; Tani, T. Hyperthermic intraperitoneal chemotherapy using a combination of mitomycin C,5-fluorouracil, and oxaliplatin in patients at high risk of colorectal peritoneal metastasis: A Phase I clinical study. *Eur J Surg Oncol* **2014**, *40*, 521–528, doi:10.1016/j.ejso.2013.12.005.
19. Shimizu, T.; Murata, S.; Sonoda, H.; Mekata, E.; Ohta, H.; Takebayashi, K.; Miyake, T.; Tani, T. Hyperthermic intraperitoneal chemotherapy with mitomycin C and 5-fluorouracil in patients at high risk of peritoneal metastasis from colorectal cancer: A preliminary clinical study. *Mol Clin Oncol* **2014**, *2*, 399–404, doi:10.3892/mco.2014.244.
20. Ceelen, W.; Van Nieuwenhove, Y.; Putte, D.V.; Pattyn, P. Neoadjuvant chemotherapy with bevacizumab may improve outcome after cytoreduction and hyperthermic intraperitoneal chemoperfusion (HIPEC) for colorectal carcinomatosis. *Ann Surg Oncol* **2014**, *21*, 3023–3028, doi:10.1245/s10434-014-3713-7.
21. Baratti, D.; Kusamura, S.; Iusco, D.; Bonomi, S.; Grassi, A.; Virzi, S.; Leo, E.; Deraco, M. Postoperative complications after cytoreductive surgery and hyperthermic intraperitoneal chemotherapy affect long-term outcome of patients with peritoneal metastases from colorectal cancer: a two-center study of 101 patients. *Dis Colon Rectum* **2014**, *57*, 858–868, doi:10.1097/DCR.0000000000000149.
22. Shen, P.; Thomas, C.R.; Fenstermaker, J.; Aklilu, M.; McCoy, T.P.; Levine, E.A. Phase II trial of adjuvant oral thalidomide following cytoreductive surgery and hyperthermic intraperitoneal chemotherapy for peritoneal surface disease from colorectal/appendiceal cancer. *J Gastrointest Cancer* **2014**, *45*, 268–275, doi:10.1007/s12029-014-9578-y.
23. Huang, C.Q.; Feng, J.P.; Yang, X.J.; Li, Y. Cytoreductive surgery plus hyperthermic intraperitoneal chemotherapy improves survival of patients with peritoneal carcinomatosis from colorectal cancer: a case-control study from a Chinese center. *J Surg Oncol* **2014**, *109*, 730–739, doi:10.1002/jso.23545.
24. Virzi, S.; Iusco, D.; Baratti, D.; Bonomi, S.; Grassi, A.; Kusamura, S.; Deraco, M. Pilot study of adjuvant hyperthermic intraperitoneal chemotherapy in patients with colorectal cancer at high risk for the development of peritoneal metastases. *Tumori* **2013**, *99*, 589–595, doi:10.1700/1377.15307.
25. Teo, M.C.; Tan, G.H.; Tham, C.K.; Lim, C.; Soo, K.C. Cytoreductive surgery and hyperthermic intraperitoneal chemotherapy in Asian patients: 100 consecutive patients in a single institution. *Ann Surg Oncol* **2013**, *20*, 2968–2974, doi:10.1245/s10434-013-2947-0.
26. Iversen, L.H.; Rasmussen, P.C.; Hagemann-Madsen, R.; Laurberg, S. Cytoreductive surgery and hyperthermic intraperitoneal chemotherapy for peritoneal carcinomatosis: the Danish experience. *Colorectal Dis* **2013**, *15*, e365-372, doi:10.1111/codi.12185.
27. Harrison, L.E.; Tiesi, G.; Razavi, R.; Wang, C.C. A phase I trial of thermal sensitization using induced oxidative stress in the context of HIPEC. *Ann Surg Oncol* **2013**, *20*, 1843–1850, doi:10.1245/s10434-013-2874-0.
28. Hompes, D.; D'Hoore, A.; Van Cutsem, E.; Fieuws, S.; Ceelen, W.; Peeters, M.; Van der Speeten, K.; Bertrand, C.; Legendre, H.; Kerger, J. The treatment of peritoneal carcinomatosis of colorectal cancer with complete cytoreductive surgery and hyperthermic intraperitoneal peroperative chemotherapy (HIPEC) with oxaliplatin: a Belgian multicentre prospective phase II clinical study. *Ann Surg Oncol* **2012**, *19*, 2186–2194, doi:10.1245/s10434-012-2264-z.

29. Cotte, E.; Passot, G.; Tod, M.; Bakrin, N.; Gilly, F.N.; Steghens, A.; Mohamed, F.; Glehen, O. Closed abdomen hyperthermic intraperitoneal chemotherapy with irinotecan and mitomycin C: a phase I study. *Ann Surg Oncol* **2011**, *18*, 2599–2603, doi:10.1245/s10434-011-1651-1.
30. Cavaliere, F.; De Simone, M.; Virzi, S.; Deraco, M.; Rossi, C.R.; Garofalo, A.; Di Filippo, F.; Giannarelli, D.; Vaira, M.; Valle, M., et al. Prognostic factors and oncologic outcome in 146 patients with colorectal peritoneal carcinomatosis treated with cytoreductive surgery combined with hyperthermic intraperitoneal chemotherapy: Italian multicenter study S.I.T.I.L.O. *Eur J Surg Oncol* **2011**, *37*, 148–154, doi:10.1016/j.ejso.2010.10.014.
31. Chua, T.C.; Morris, D.L.; Esquivel, J. Impact of the peritoneal surface disease severity score on survival in patients with colorectal cancer peritoneal carcinomatosis undergoing complete cytoreduction and hyperthermic intraperitoneal chemotherapy. *Ann Surg Oncol* **2010**, *17*, 1330–1336, doi:10.1245/s10434-009-0866-x.
32. Yang, X.J.; Li, Y.; al-shammaa Hassan, A.H.; Yang, G.L.; Liu, S.Y.; Lu, Y.L.; Zhang, J.W.; Yonemura, Y. Cytoreductive surgery plus hyperthermic intraperitoneal chemotherapy improves survival in selected patients with peritoneal carcinomatosis from abdominal and pelvic malignancies: results of 21 cases. *Ann Surg Oncol* **2009**, *16*, 345–351, doi:10.1245/s10434-008-0226-2.
33. Lanuke, K.; Mack, L.A.; Temple, W.J. Phase II study of regional treatment for peritoneal carcinomatosis. *Am J Surg* **2009**, *197*, 614–618; discussion 618, doi:10.1016/j.amjsurg.2008.12.026.
34. Chouillard, E.; Ata, T.; De Jonghe, B.; Maggiori, L.; Helmy, N.; Coscas, Y.; Outin, H. Staged laparoscopic adjuvant intraperitoneal chemohyperthermia after complete resection for locally advanced colorectal or gastric cancer: a preliminary experience. *Surg Endosc* **2009**, *23*, 363–369, doi:10.1007/s00464-008-9946-4.
35. Asero, S.; Caruso, M.; Vallone, N.; Luciani, A.G.; Lombardo, V.; Terranova, G.; Ettore, G.; Giannone, G. Cytoreductive surgery (cs) and hyperthermic intraperitoneal chemotherapy (hipec) in treatment of peritoneal surface malignances: report of a phase II clinical study. *In Vivo* **2009**, *23*, 645–647.
36. Chua, T.C.; Pelz, J.O.; Kerscher, A.; Morris, D.L.; Esquivel, J. Critical analysis of 33 patients with peritoneal carcinomatosis secondary to colorectal and appendiceal signet ring cell carcinoma. *Ann Surg Oncol* **2009**, *16*, 2765–2770, doi:10.1245/s10434-009-0536-z.
37. Verwaal, V.J.; Bruin, S.; Boot, H.; van Slooten, G.; van Tinteren, H. 8-year follow-up of randomized trial: cytoreduction and hyperthermic intraperitoneal chemotherapy versus systemic chemotherapy in patients with peritoneal carcinomatosis of colorectal cancer. *Ann Surg Oncol* **2008**, *15*, 2426–2432, doi:10.1245/s10434-008-9966-2.
38. van Leeuwen, B.L.; Graf, W.; Pahlman, L.; Mahteme, H. Swedish experience with peritonectomy and HIPEC. HIPEC in peritoneal carcinomatosis. *Ann Surg Oncol* **2008**, *15*, 745–753, doi:10.1245/s10434-007-9700-5.
39. Stewart, J.H.t.; Shen, P.; Russell, G.; Fenstermaker, J.; McWilliams, L.; Coldrun, F.M.; Levine, K.E.; Jones, B.T.; Levine, E.A. A phase I trial of oxaliplatin for intraperitoneal hyperthermic chemoperfusion for the treatment of peritoneal surface dissemination from colorectal and appendiceal cancers. *Ann Surg Oncol* **2008**, *15*, 2137–2145, doi:10.1245/s10434-008-9967-1.
40. Harrison, L.E.; Bryan, M.; Pliner, L.; Saunders, T. Phase I trial of pegylated liposomal doxorubicin with hyperthermic intraperitoneal chemotherapy in patients undergoing cytoreduction for advanced intra-abdominal malignancy. *Ann Surg Oncol* **2008**, *15*, 1407–1413, doi:10.1245/s10434-007-9718-8.
41. Gusani, N.J.; Cho, S.W.; Colovos, C.; Seo, S.; Franko, J.; Richard, S.D.; Edwards, R.P.; Brown, C.K.; Holtzman, M.P.; Zeh, H.J., et al. Aggressive surgical management of peritoneal carcinomatosis with low mortality in a high-volume tertiary cancer center. *Ann Surg Oncol* **2008**, *15*, 754–763, doi:10.1245/s10434-007-9701-4.
42. Elias, D.; Pocard, M.; Goere, D. HIPEC with oxaliplatin in the treatment of peritoneal carcinomatosis of colorectal origin. *Cancer Treat Res* **2007**, *134*, 303–318.
43. Piso, P.; Dahlke, M.H.; Ghali, N.; Iesalnieks, I.; Loss, M.; Popp, F.; von Breitenbuch, P.; Agha, A.; Lang, S.A.; Kullmann, F., et al. Multimodality treatment of peritoneal carcinomatosis from colorectal cancer: first results of a new German centre for peritoneal surface malignancies. *Int J Colorectal Dis* **2007**, *22*, 1295–1300, doi:10.1007/s00384-007-0313-z.
44. Zanon, C.; Bortolini, M.; Chiappino, I.; Simone, P.; Bruno, F.; Gaglia, P.; Airoldi, M.; Deriu, L.; Mashiah, A. Cytoreductive surgery combined with intraperitoneal chemohyperthermia for the treatment of advanced colon cancer. *World J Surg* **2006**, *30*, 2025–2032, doi:10.1007/s00268-005-0486-y.

45. Yan, T.D.; Chu, F.; Links, M.; Kam, P.C.; Glenn, D.; Morris, D.L. Cytoreductive surgery and perioperative intraperitoneal chemotherapy for peritoneal carcinomatosis from colorectal carcinoma: non-mucinous tumour associated with an improved survival. *Eur J Surg Oncol* **2006**, *32*, 1119–1124, doi:10.1016/j.ejso.2006.06.007.
46. Elias, D.; Raynard, B.; Farkhondeh, F.; Goere, D.; Rouquie, D.; Ciuchendea, R.; Pocard, M.; Ducreux, M. Peritoneal carcinomatosis of colorectal origin. *Gastroenterol Clin Biol* **2006**, *30*, 1200–1204.
47. Elias, D.; Raynard, B.; Bonnay, M.; Pocard, M. Heated intra-operative intraperitoneal oxaliplatin alone and in combination with intraperitoneal irinotecan: Pharmacologic studies. *Eur J Surg Oncol* **2006**, *32*, 607–613, doi:10.1016/j.ejso.2006.03.004.
48. da Silva, R.G.; Sugarbaker, P.H. Analysis of prognostic factors in seventy patients having a complete cytoreduction plus perioperative intraperitoneal chemotherapy for carcinomatosis from colorectal cancer. *J Am Coll Surg* **2006**, *203*, 878–886, doi:10.1016/j.jamcollsurg.2006.08.024.
49. Verwaal, V.J.; van Ruth, S.; Witkamp, A.; Boot, H.; van Slooten, G.; Zoetmulder, F.A. Long-term survival of peritoneal carcinomatosis of colorectal origin. *Ann Surg Oncol* **2005**, *12*, 65–71, doi:10.1007/s10434-004-1167-z.
50. Kecmanovic, D.M.; Pavlov, M.J.; Ceranic, M.S.; Sepetkovski, A.V.; Kovacevic, P.A.; Stamenkovic, A.B. Treatment of peritoneal carcinomatosis from colorectal cancer by cytoreductive surgery and hyperthermic perioperative intraperitoneal chemotherapy. *Eur J Surg Oncol* **2005**, *31*, 147–152, doi:10.1016/j.ejso.2004.09.021.
51. Verwaal, V.J.; van Tinteren, H.; van Ruth, S.; Zoetmulder, F.A. Predicting the survival of patients with peritoneal carcinomatosis of colorectal origin treated by aggressive cytoreduction and hyperthermic intraperitoneal chemotherapy. *Br J Surg* **2004**, *91*, 739–746, doi:10.1002/bjs.4516.
52. Elias, D.; Sideris, L.; Pocard, M.; Ede, C.; Ben Hassouna, D.; Ducreux, M.; Boige, V.; Cote, J.F.; Lasser, P. Efficacy of intraperitoneal chemohyperthermia with oxaliplatin in colorectal peritoneal carcinomatosis. Preliminary results in 24 patients. *Ann Oncol* **2004**, *15*, 781–785.
53. Verwaal, V.J.; van Ruth, S.; de Bree, E.; van Sloothen, G.W.; van Tinteren, H.; Boot, H.; Zoetmulder, F.A. Randomized trial of cytoreduction and hyperthermic intraperitoneal chemotherapy versus systemic chemotherapy and palliative surgery in patients with peritoneal carcinomatosis of colorectal cancer. *J Clin Oncol* **2003**, *21*, 3737–3743, doi:10.1200/JCO.2003.04.187.
54. Pilati, P.; Mocellin, S.; Rossi, C.R.; Foletto, M.; Campana, L.; Nitti, D.; Lise, M. Cytoreductive surgery combined with hyperthermic intraperitoneal intraoperative chemotherapy for peritoneal carcinomatosis arising from colon adenocarcinoma. *Ann Surg Oncol* **2003**, *10*, 508–513.
55. Glehen, O.; Mithieux, F.; Osinsky, D.; Beaujard, A.C.; Freyer, G.; Guertsch, P.; Francois, Y.; Peyrat, P.; Panteix, G.; Vignal, J., et al. Surgery combined with peritonectomy procedures and intraperitoneal chemohyperthermia in abdominal cancers with peritoneal carcinomatosis: a phase II study. *J Clin Oncol* **2003**, *21*, 799–806, doi:10.1200/JCO.2003.06.139.
56. Fu, Q.G.; Meng, F.D.; Shen, X.D.; Guo, R.X. Efficacy of intraperitoneal thermochemotherapy and immunotherapy in intraperitoneal recurrence after gastrointestinal cancer resection. *World J Gastroenterol* **2002**, *8*, 1019–1022.
57. Elias, D.; Bonnay, M.; Puizillou, J.M.; Antoun, S.; Demirdjian, S.; El, O.A.; Pignon, J.P.; Drouard-Troalen, L.; Ouellet, J.F.; Ducreux, M. Heated intra-operative intraperitoneal oxaliplatin after complete resection of peritoneal carcinomatosis: pharmacokinetics and tissue distribution. *Ann Oncol* **2002**, *13*, 267–272.
58. Witkamp, A.J.; de Bree, E.; Kaag, M.M.; Boot, H.; Beijnen, J.H.; van Slooten, G.W.; van Coevorden, F.; Zoetmulder, F.A. Extensive cytoreductive surgery followed by intra-operative hyperthermic intraperitoneal chemotherapy with mitomycin-C in patients with peritoneal carcinomatosis of colorectal origin. *Eur J Cancer* **2001**, *37*, 979–984.
59. Beaujard, A.C.; Glehen, O.; Caillot, J.L.; Francois, Y.; Bienvenu, J.; Panteix, G.; Garbit, F.; Grandclement, E.; Vignal, J.; Gilly, F.N. Intraperitoneal chemohyperthermia with mitomycin C for digestive tract cancer patients with peritoneal carcinomatosis. *Cancer* **2000**, *88*, 2512–2519.
60. Tsiftsis, D.; de Bree, E.; Romanos, J.; Petrou, A.; Sanidas, E.; Askoxylakis, J.; Zervos, K.; Michaloudis, D. Peritoneal expansion by artificially produced ascites during perfusion chemotherapy. *Arch Surg* **1999**, *134*, 545–549; discussion 550.
61. Gilly, F.N.; Beaujard, A.; Glehen, O.; Grandclement, E.; Caillot, J.L.; Francois, Y.; Sadeghi-Looyeh, B.; Gueugniaud, P.Y.; Garbit, F.; Benoit, M., et al. Peritonectomy combined with intraperitoneal

chemohyperthermia in abdominal cancer with peritoneal carcinomatosis: phase I-II study. *Anticancer Res* **1999**, *19*, 2317–2321.

62. Yarema, R.; Fetsych, T.; Volodko, N.; capital O, C.M.; Petronchak, O.; Huley, R.; Mylyan, Y.; Glehen, O. Evaluation of the peritoneal surface disease severity score (PSDSS) in ovarian cancer patients undergoing cytoreductive surgery and HIPEC: Two pathogenetic types based study. *J Surg Oncol* **2018**, 10.1002/jso.25087, doi:10.1002/jso.25087.
63. van Driel, W.J.; Koole, S.N.; Sikorska, K.; Schagen van Leeuwen, J.H.; Schreuder, H.W.R.; Hermans, R.H.M.; de Hingh, I.; van der Velden, J.; Arts, H.J.; Massuger, L., et al. Hyperthermic Intraperitoneal Chemotherapy in Ovarian Cancer. *N Engl J Med* **2018**, *378*, 230–240, doi:10.1056/NEJMoa1708618.
64. Pavlov, M.J.; Ceranic, M.S.; Latincic, S.M.; Sabljak, P.V.; Kecmanovic, D.M.; Sugarbaker, P.H. Cytoreductive surgery and hyperthermic intraperitoneal chemotherapy for the treatment of advanced epithelial and recurrent ovarian carcinoma: a single center experience. *Int J Hyperthermia* **2018**, *34*, 564–569, doi:10.1080/02656736.2017.1371341.
65. Batista, T.P.; Carneiro, V.C.G.; Tancredi, R.; Teles, A.L.B.; Badiglian-Filho, L.; Leao, C.S. Neoadjuvant chemotherapy followed by fast-track cytoreductive surgery plus short-course hyperthermic intraperitoneal chemotherapy (HIPEC) in advanced ovarian cancer: preliminary results of a promising all-in-one approach. *Cancer Manag Res* **2017**, *9*, 869–878, doi:10.2147/CMAR.S153327.
66. Sun, J.H.; Ji, Z.H.; Yu, Y.; Wu, H.T.; Huang, C.Q.; Zhang, Q.; Yang, X.J.; Yonemura, Y.; Li, Y. Cytoreductive Surgery plus Hyperthermic Intraperitoneal Chemotherapy to Treat Advanced/Recurrent Epithelial Ovarian Cancer: Results from a Retrospective Study on Prospectively Established Database. *Transl Oncol* **2016**, *9*, 130–138, doi:10.1016/j.tranon.2016.02.002.
67. Sanchez-Garcia, S.; Villarejo-Campos, P.; Padilla-Valverde, D.; Amo-Salas, M.; Martin-Fernandez, J. Intraperitoneal chemotherapy hyperthermia (HIPEC) for peritoneal carcinomatosis of ovarian cancer origin by fluid and CO2 recirculation using the closed abdomen technique (PRS-1.0 Combat): A clinical pilot study. *Int J Hyperthermia* **2016**, *32*, 488–495, doi:10.3109/02656736.2016.1152515.
68. Petrillo, M.; De Iaco, P.; Cianci, S.; Perrone, M.; Costantini, B.; Ronsini, C.; Scambia, G.; Fagotti, A. Long-Term Survival for Platinum-Sensitive Recurrent Ovarian Cancer Patients Treated with Secondary Cytoreductive Surgery Plus Hyperthermic Intraperitoneal Chemotherapy (HIPEC). *Ann Surg Oncol* **2016**, *23*, 1660–1665, doi:10.1245/s10434-015-5050-x.
69. Hayes-Jordan, A.; Lopez, C.; Green, H.L.; Xiao, L.C.; Huh, W.; Herzog, C.E. Cytoreductive surgery (CRS) and hyperthermic intraperitoneal chemotherapy (HIPEC) in pediatric ovarian tumors: a novel treatment approach. *Pediatr Surg Int* **2016**, *32*, 71–73, doi:10.1007/s00383-015-3814-9.
70. Gouy, S.; Ferron, G.; Glehen, O.; Bayar, A.; Marchal, F.; Pomel, C.; Quenet, F.; Bereder, J.M.; Le Deley, M.C.; Morice, P. Results of a multicenter phase I dose-finding trial of hyperthermic intraperitoneal cisplatin after neoadjuvant chemotherapy and complete cytoreductive surgery and followed by maintenance bevacizumab in initially unresectable ovarian cancer. *Gynecol Oncol* **2016**, *142*, 237–242, doi:10.1016/j.ygyno.2016.05.032.
71. D'Hondt, V.; Goffin, F.; Roca, L.; Dresse, D.; Leroy, C.; Kerger, J.; Cordier, L.; de Forges, H.; Veys, I.; Liberale, G. Interval Cytoreductive Surgery and Hyperthermic Intraperitoneal Chemotherapy in First-Line Treatment for Advanced Ovarian Carcinoma: A Feasibility Study. *Int J Gynecol Cancer* **2016**, *26*, 912–917, doi:10.1097/IGC.0000000000000696.
72. Baiocchi, G.; Ferreira, F.O.; Mantoan, H.; da Costa, A.A.; Faloppa, C.C.; Kumagai, L.Y.; de Mello, C.A.; Takahashi, R.M.; Nakagawa, W.T.; Aguiar, S., Jr., et al. Hyperthermic Intraperitoneal Chemotherapy after Secondary Cytoreduction in Epithelial Ovarian Cancer: A Single-center Comparative Analysis. *Ann Surg Oncol* **2016**, *23*, 1294–1301, doi:10.1245/s10434-015-4991-4.
73. Ba, M.; Long, H.; Zhang, X.; Tang, Y.; Wu, Y.; Wang, S.; Yan, Z.; Zhang, B.; Cui, S. Hyperthermic Intraperitoneal Perfusion Chemotherapy and Cytoreductive Surgery for Controlling Malignant Ascites From Ovarian Cancer. *Int J Gynecol Cancer* **2016**, *26*, 1571–1579, doi:10.1097/IGC.0000000000000809.
74. Abramian, A.; Zivanovic, O.; Kuhn, W.; Weber, S.; Schaefer, N.; Keyver-Paik, M.D.; Kiefer, N. Introducing Hyperthermic Intraperitoneal Chemotherapy into Gynecological Oncology Practice - Feasibility and Safety Considerations: Single-Center Experience. *Oncol Res Treat* **2016**, *39*, 178–184, doi:10.1159/000445180.
75. Zivanovic, O.; Abramian, A.; Kullmann, M.; Fuhrmann, C.; Coch, C.; Hoeller, T.; Ruehs, H.; Keyver-Paik, M.D.; Rudlowski, C.; Weber, S., et al. HIPEC ROC I: a phase I study of cisplatin administered as hyperthermic

intraoperative intraperitoneal chemoperfusion followed by postoperative intravenous platinum-based chemotherapy in patients with platinum-sensitive recurrent epithelial ovarian cancer. *Int J Cancer* **2015**, *136*, 699–708, doi:10.1002/ijc.29011.

76. Topgul, K.; Cetinkaya, M.B.; Cigdem Arslan, N.; Gul, M.K.; Can, M.; Gursel, M.F.; Erdem, D.; Malazgirt, Z. Cytoreductive surgery (SRC) and hyperthermic intraperitoneal chemotherapy (HIPEC) for treatment of peritoneal carcinomatosis: Our initial experience and technical details. *Ulus Cerrahi Derg* **2015**, *31*, 138–147, doi:10.5152/UCD.2015.2990.
77. Spiliotis, J.; Halkia, E.; Lianos, E.; Kalantzi, N.; Grivas, A.; Efstathiou, E.; Giassas, S. Cytoreductive surgery and HIPEC in recurrent epithelial ovarian cancer: a prospective randomized phase III study. *Ann Surg Oncol* **2015**, *22*, 1570–1575, doi:10.1245/s10434-014-4157-9.
78. Rettenmaier, M.A.; Mendivil, A.A.; Abaid, L.N.; Brown, J.V., 3rd; Micha, J.P.; Wilcox, A.M.; Goldstein, B.H. The feasibility of administering varying high-dose consolidation hyperthermic intraperitoneal chemotherapy with carboplatin in the treatment of ovarian carcinoma. *Arch Gynecol Obstet* **2015**, *291*, 1381–1386, doi:10.1007/s00404-014-3590-0.
79. Fagotti, A.; Costantini, B.; Gallotta, V.; Cianci, S.; Ronsini, C.; Petrillo, M.; Pacciani, M.; Scambia, G.; Fanfani, F. Minimally invasive secondary cytoreduction plus HIPEC versus open surgery plus HIPEC in isolated relapse from ovarian cancer: a retrospective cohort study on perioperative outcomes. *J Minim Invasive Gynecol* **2015**, *22*, 428–432, doi:10.1016/j.jmig.2014.11.008.
80. Coccolini, F.; Campanati, L.; Catena, F.; Ceni, V.; Ceresoli, M.; Jimenez Cruz, J.; Lotti, M.; Magnone, S.; Napoli, J.; Rossetti, D., et al. Hyperthermic intraperitoneal chemotherapy with cisplatin and paclitaxel in advanced ovarian cancer: a multicenter prospective observational study. *J Gynecol Oncol* **2015**, *26*, 54–61, doi:10.3802/jgo.2015.26.1.54.
81. Le Brun, J.F.; Campion, L.; Berton-Rigaud, D.; Lorimier, G.; Marchal, F.; Ferron, G.; Oger, A.S.; Dravet, F.; Jaffre, I.; Classe, J.M. Survival benefit of hyperthermic intraperitoneal chemotherapy for recurrent ovarian cancer: a multi-institutional case control study. *Ann Surg Oncol* **2014**, *21*, 3621–3627, doi:10.1245/s10434-014-3693-7.
82. Ba, M.; Long, H.; Zhang, X.; Tang, Y.; Wu, Y.; Yu, F.; Wang, S.; Cui, S. Different sequential approaches of cytoreductive surgery and hyperthermic intraperitoneal chemotherapy in treating ovarian cancer with malignant ascites. *J Cancer Res Clin Oncol* **2014**, *140*, 1497–1506, doi:10.1007/s00432-014-1692-5.
83. Warschkow, R.; Tarantino, I.; Lange, J.; Muller, S.A.; Schmied, B.M.; Zund, M.; Steffen, T. Does hyperthermic intraoperative chemotherapy lead to improved outcomes in patients with ovarian cancer? A single center cohort study in 111 consecutive patients. *Patient Saf Surg* **2012**, *6*, 12, doi:10.1186/1754-9493-6-12.
84. Tentes, A.A.; Kakolyris, S.; Kyziridis, D.; Karamveri, C. Cytoreductive surgery combined with hyperthermic intraperitoneal intraoperative chemotherapy in the treatment of advanced epithelial ovarian cancer. *J Oncol* **2012**, *2012*, 358341, doi:10.1155/2012/358341.
85. Deraco, M.; Virzi, S.; Iusco, D.R.; Puccio, F.; Macri, A.; Famulari, C.; Solazzo, M.; Bonomi, S.; Grassi, A.; Baratti, D., et al. Secondary cytoreductive surgery and hyperthermic intraperitoneal chemotherapy for recurrent epithelial ovarian cancer: a multi-institutional study. *BJOG* **2012**, *119*, 800–809, doi:10.1111/j.1471-0528.2011.03207.x.
86. Ansaloni, L.; Agnoletti, V.; Amadori, A.; Catena, F.; Cavaliere, D.; Coccolini, F.; De Iaco, P.; Di Battista, M.; Framarini, M.; Gazzotti, F., et al. Evaluation of extensive cytoreductive surgery and hyperthermic intraperitoneal chemotherapy (HIPEC) in patients with advanced epithelial ovarian cancer. *Int J Gynecol Cancer* **2012**, *22*, 778–785, doi:10.1097/IGC.0b013e31824d836c.
87. Pomel, C.; Ferron, G.; Lorimier, G.; Rey, A.; Lhomme, C.; Classe, J.M.; Bereder, J.M.; Quenet, F.; Meeus, P.; Marchal, F., et al. Hyperthermic intra-peritoneal chemotherapy using oxaliplatin as consolidation therapy for advanced epithelial ovarian carcinoma. Results of a phase II prospective multicentre trial. CHIPOVAC study. *Eur J Surg Oncol* **2010**, *36*, 589–593, doi:10.1016/j.ejso.2010.04.005.
88. Pavlov, M.J.; Kovacevic, P.A.; Ceranic, M.S.; Stamenkovic, A.B.; Ivanovic, A.M.; Kecmanovic, D.M. Cytoreductive surgery and modified heated intraoperative intraperitoneal chemotherapy (HIPEC) for advanced and recurrent ovarian cancer -- 12-year single center experience. *Eur J Surg Oncol* **2009**, *35*, 1186–1191, doi:10.1016/j.ejso.2009.03.004.

89. Lim, M.C.; Kang, S.; Choi, J.; Song, Y.J.; Park, S.; Seo, S.S.; Park, S.Y. Hyperthermic intraperitoneal chemotherapy after extensive cytoreductive surgery in patients with primary advanced epithelial ovarian cancer: interim analysis of a phase II study. *Ann Surg Oncol* **2009**, *16*, 993–1000, doi:10.1245/s10434-008-0299-y.
90. Fagotti, A.; Paris, I.; Grimolizzi, F.; Fanfani, F.; Vizzielli, G.; Naldini, A.; Scambia, G. Secondary cytoreduction plus oxaliplatin-based HIPEC in platinum-sensitive recurrent ovarian cancer patients: a pilot study. *Gynecol Oncol* **2009**, *113*, 335–340, doi:10.1016/j.ygyno.2009.03.004.
91. Di Giorgio, A.; Naticchioni, E.; Biacchi, D.; Sibio, S.; Accarpio, F.; Rocco, M.; Tarquini, S.; Di Seri, M.; Ciardi, A.; Montruccoli, D., et al. Cytoreductive surgery (peritonectomy procedures) combined with hyperthermic intraperitoneal chemotherapy (HIPEC) in the treatment of diffuse peritoneal carcinomatosis from ovarian cancer. *Cancer* **2008**, *113*, 315–325, doi:10.1002/cncr.23553.
92. de Bree, E.; Rosing, H.; Filis, D.; Romanos, J.; Melisssourgaki, M.; Daskalakis, M.; Pilatou, M.; Sanidas, E.; Taflampas, P.; Kalbakis, K., et al. Cytoreductive surgery and intraoperative hyperthermic intraperitoneal chemotherapy with paclitaxel: a clinical and pharmacokinetic study. *Ann Surg Oncol* **2008**, *15*, 1183–1192, doi:10.1245/s10434-007-9792-y.
93. Lentz, S.S.; Miller, B.E.; Kucera, G.L.; Levine, E.A. Intraperitoneal hyperthermic chemotherapy using carboplatin: a phase I analysis in ovarian carcinoma. *Gynecol Oncol* **2007**, *106*, 207–210, doi:10.1016/j.ygyno.2007.03.022.
94. Kusamura, S.; Younan, R.; Baratti, D.; Costanzo, P.; Favaro, M.; Gavazzi, C.; Deraco, M. Cytoreductive surgery followed by intraperitoneal hyperthermic perfusion: analysis of morbidity and mortality in 209 peritoneal surface malignancies treated with closed abdomen technique. *Cancer* **2006**, *106*, 1144–1153, doi:10.1002/cncr.21708.
95. Gori, J.; Castano, R.; Toziano, M.; Habich, D.; Staringer, J.; De Quiros, D.G.; Felci, N. Intraperitoneal hyperthermic chemotherapy in ovarian cancer. *Int J Gynecol Cancer* **2005**, *15*, 233–239, doi:10.1111/j.1525-1438.2005.15209.x.
96. Zanon, C.; Clara, R.; Chiappino, I.; Bortolini, M.; Cornaglia, S.; Simone, P.; Bruno, F.; De Riu, L.; Airoldi, M.; Pedani, F. Cytoreductive surgery and intraperitoneal chemohyperthermia for recurrent peritoneal carcinomatosis from ovarian cancer. *World J Surg* **2004**, *28*, 1040–1045, doi:10.1007/s00268-004-7461-x.
97. Ryu, K.S.; Kim, J.H.; Ko, H.S.; Kim, J.W.; Ahn, W.S.; Park, Y.G.; Kim, S.J.; Lee, J.M. Effects of intraperitoneal hyperthermic chemotherapy in ovarian cancer. *Gynecol Oncol* **2004**, *94*, 325–332, doi:10.1016/j.ygyno.2004.05.044.
98. Kusamura, S.; Deraco, M.; Baratti, D.; Inglese, M.G.; Costanzo, P.; Favaro, M.; Manzi, R.; Gavazzi, C. Cytoreductive surgery followed by intra peritoneal hyperthermic perfusion in the treatment of peritoneal surface malignancies: morbidity and mortality with closed abdomen technique. *J Exp Clin Cancer Res* **2003**, *22*, 207–212.
99. Hager, E.D.; Dziambor, H.; Hohmann, D.; Muhe, N.; Strama, H. Intraperitoneal hyperthermic perfusion chemotherapy of patients with chemotherapy-resistant peritoneal disseminated ovarian cancer. *Int J Gynecol Cancer* **2001**, *11 Suppl 1*, 57–63.
100. Deraco, M.; Rossi, C.R.; Pennacchioli, E.; Guadagni, S.; Somers, D.C.; Santoro, N.; Raspagliesi, F.; Kusamura, S.; Vaglini, M. Cytoreductive surgery followed by intraperitoneal hyperthermic perfusion in the treatment of recurrent epithelial ovarian cancer: a phase II clinical study. *Tumori* **2001**, *87*, 120–126.
101. van der Vange, N.; van Goethem, A.R.; Zoetmulder, F.A.; Kaag, M.M.; van de Vaart, P.J.; ten Bokkel Huinink, W.W.; Beijnen, J.H. Extensive cytoreductive surgery combined with intra-operative intraperitoneal perfusion with cisplatin under hyperthermic conditions (OVHIPEC) in patients with recurrent ovarian cancer: a feasibility pilot. *Eur J Surg Oncol* **2000**, *26*, 663–668, doi:10.1053/ejso.2000.0978.
102. Tu, Y.; Tian, Y.; Wu, Y.; Cui, S. Clinical significance of heat shock proteins in gastric cancer following hyperthermia stress: Indications for hyperthermic intraperitoneal chemoperfusion therapy. *Oncol Lett* **2018**, *15*, 9385–9391, doi:10.3892/ol.2018.8508.
103. van der Kaaij, R.T.; Braam, H.J.; Boot, H.; Los, M.; Cats, A.; Grootscholten, C.; Schellens, J.H.; Aalbers, A.G.; Huitema, A.D.; Knibbe, C.A., et al. Treatment of Peritoneal Dissemination in Stomach Cancer Patients With Cytoreductive Surgery and Hyperthermic Intraperitoneal Chemotherapy (HIPEC): Rationale and Design of the PERISCOPE Study. *JMIR Res Protoc* **2017**, *6*, e136, doi:10.2196/resprot.7790.

104. Topal, B.; Demey, K.; Topal, H.; Jaekers, J.; Van Cutsem, E.; Vandecaveye, V.; Sagaert, X.; Prenen, H. Cytoreductive surgery and Hyperthermic intra-operative peritoneal chemotherapy with Cisplatin for gastric peritoneal Carcinomatosis Monocentric phase-2 nonrandomized prospective clinical trial. *BMC Cancer* **2017**, *17*, 771, doi:10.1186/s12885-017-3730-6.
105. Murata, S.; Yamamoto, H.; Naitoh, H.; Yamaguchi, T.; Kaida, S.; Shimizu, T.; Shiomi, H.; Naka, S.; Tani, T.; Tani, M. Feasibility and safety of hyperthermic intraperitoneal chemotherapy using 5-fluorouracil combined with cisplatin and mitomycin C in patients undergoing gastrectomy for advanced gastric cancer. *J Surg Oncol* **2017**, *116*, 1159–1165, doi:10.1002/jso.24771.
106. Wu, H.T.; Peng, K.W.; Ji, Z.H.; Sun, J.H.; Zhang, Q.; Yang, X.J.; Huang, C.Q.; Li, Y. Cytoreductive surgery plus hyperthermic intraperitoneal chemotherapy with lobaplatin and docetaxel to treat synchronous peritoneal carcinomatosis from gastric cancer: Results from a Chinese center. *Eur J Surg Oncol* **2016**, *42*, 1024–1034, doi:10.1016/j.ejso.2016.04.053.
107. Boerner, T.; Graichen, A.; Jeiter, T.; Zemann, F.; Renner, P.; Marz, L.; Soeder, Y.; Schlitt, H.J.; Piso, P.; Dahlke, M.H. CRS-HIPEC Prolongs Survival but is Not Curative for Patients with Peritoneal Carcinomatosis of Gastric Cancer. *Ann Surg Oncol* **2016**, *23*, 3972–3977, doi:10.1245/s10434-016-5306-0.
108. Yarema, R.R.; Ohorchak, M.A.; Zubarev, G.P.; Mylyan, Y.P.; Oliynyk, Y.Y.; Zubarev, M.G.; Gyrya, P.I.; Kovalchuk, Y.J.; Safiyan, V.I.; Fetsych, T.G. Hyperthermic intraperitoneal chemoperfusion in combined treatment of locally advanced and disseminated gastric cancer: results of a single-centre retrospective study. *Int J Hyperthermia* **2014**, *30*, 159–165, doi:10.3109/02656736.2014.893451.
109. Glehen, O.; Passot, G.; Villeneuve, L.; Vaudoyer, D.; Bin-Dorel, S.; Boschetti, G.; Piaton, E.; Garofalo, A. GASTRICHIP: D2 resection and hyperthermic intraperitoneal chemotherapy in locally advanced gastric carcinoma: a randomized and multicenter phase III study. *BMC Cancer* **2014**, *14*, 183, doi:10.1186/1471-2407-14-183.
110. Hultman, B.; Lind, P.; Glimelius, B.; Sundbom, M.; Nygren, P.; Haglund, U.; Mahteme, H. Phase II study of patients with peritoneal carcinomatosis from gastric cancer treated with preoperative systemic chemotherapy followed by peritonectomy and intraperitoneal chemotherapy. *Acta Oncol* **2013**, *52*, 824–830, doi:10.3109/0284186X.2012.702925.
111. Hultman, B.; Lundkvist, J.; Glimelius, B.; Nygren, P.; Mahteme, H. Costs and clinical outcome of neoadjuvant systemic chemotherapy followed by cytoreductive surgery and hyperthermic intraperitoneal chemotherapy in peritoneal carcinomatosis from gastric cancer. *Acta Oncol* **2012**, *51*, 112–121, doi:10.3109/0284186X.2011.594809.
112. Yang, X.J.; Huang, C.Q.; Suo, T.; Mei, L.J.; Yang, G.L.; Cheng, F.L.; Zhou, Y.F.; Xiong, B.; Yonemura, Y.; Li, Y. Cytoreductive surgery and hyperthermic intraperitoneal chemotherapy improves survival of patients with peritoneal carcinomatosis from gastric cancer: final results of a phase III randomized clinical trial. *Ann Surg Oncol* **2011**, *18*, 1575–1581, doi:10.1245/s10434-011-1631-5.
113. Evers, D.J.; Smeenk, R.M.; Bottenberg, P.D.; van Werkhoven, E.D.; Boot, H.; Verwaal, V.J. Effect of preservation of the right gastro-epiploic artery on delayed gastric emptying after cytoreductive surgery and HIPEC: a randomized clinical trial. *Eur J Surg Oncol* **2011**, *37*, 162–167, doi:10.1016/j.ejso.2010.12.005.
114. Yang, X.J.; Li, Y.; Yonemura, Y. Cytoreductive surgery plus hyperthermic intraperitoneal chemotherapy to treat gastric cancer with ascites and/or peritoneal carcinomatosis: Results from a Chinese center. *J Surg Oncol* **2010**, *101*, 457–464, doi:10.1002/jso.21519.
115. Li, C.; Yan, M.; Chen, J.; Xiang, M.; Zhu, Z.G.; Yin, H.R.; Lin, Y.Z. Surgical resection with hyperthermic intraperitoneal chemotherapy for gastric cancer patients with peritoneal dissemination. *J Surg Oncol* **2010**, *102*, 361–365, doi:10.1002/jso.21628.
116. Piso, P.; Slowik, P.; Popp, F.; Dahlke, M.H.; Glockzin, G.; Schlitt, H.J. Safety of gastric resections during cytoreductive surgery and hyperthermic intraperitoneal chemotherapy for peritoneal carcinomatosis. *Ann Surg Oncol* **2009**, *16*, 2188–2194, doi:10.1245/s10434-009-0478-5.
117. Zhu, Z.G.; Tang, R.; Yan, M.; Chen, J.; Yang, Q.M.; Li, C.; Yao, X.X.; Zhang, J.; Yin, H.R.; Lin, Y.Z. Efficacy and safety of intraoperative peritoneal hyperthermic chemotherapy for advanced gastric cancer patients with serosal invasion. A long-term follow-up study. *Dig Surg* **2006**, *23*, 93–102, doi:10.1159/000093778.

118. Hall, J.J.; Loggie, B.W.; Shen, P.; Beamer, S.; Douglas Case, L.; McQuellon, R.; Geisinger, K.R.; Levine, E.A. Cytoreductive surgery with intraperitoneal hyperthermic chemotherapy for advanced gastric cancer. *J Gastrointest Surg* **2004**, *8*, 454–463, doi:10.1016/j.gassur.2003.12.014.
119. Glehen, O.; Schreiber, V.; Cotte, E.; Sayag-Beaujard, A.C.; Osinsky, D.; Freyer, G.; Francois, Y.; Vignal, J.; Gilly, F.N. Cytoreductive surgery and intraperitoneal chemohyperthermia for peritoneal carcinomatosis arising from gastric cancer. *Arch Surg* **2004**, *139*, 20–26, doi:10.1001/archsurg.139.1.20.
120. Kunisaki, C.; Shimada, H.; Nomura, M.; Akiyama, H.; Takahashi, M.; Matsuda, G. Lack of efficacy of prophylactic continuous hyperthermic peritoneal perfusion on subsequent peritoneal recurrence and survival in patients with advanced gastric cancer. *Surgery* **2002**, *131*, 521–528.
121. Kern, W.; Braess, J.; Kotschofsky, M.; Samel, S.; Becker, H.; Hiddemann, W.; Schleyer, E. Application of cisplatin as intraoperative hyperthermic peritoneal lavage (IHPL) in patients with locally advanced gastric cancer: analysis of pharmacokinetics and of nephrotoxicity. *Anticancer Res* **2002**, *22*, 3099–3102.
122. Kim, J.Y.; Bae, H.S. A controlled clinical study of serosa-invasive gastric carcinoma patients who underwent surgery plus intraperitoneal hyperthermo-chemo-perfusion (IHCP). *Gastric Cancer* **2001**, *4*, 27–33, doi:10.1007/s101200100013.
123. Stephens, A.D.; Alderman, R.; Chang, D.; Edwards, G.D.; Esquivel, J.; Sebbag, G.; Steves, M.A.; Sugarbaker, P.H. Morbidity and mortality analysis of 200 treatments with cytoreductive surgery and hyperthermic intraoperative intraperitoneal chemotherapy using the coliseum technique. *Ann Surg Oncol* **1999**, *6*, 790–796.
124. Sayag-Beaujard, A.C.; Francois, Y.; Glehen, O.; Sadeghi-Looyeh, B.; Bienvenu, J.; Panteix, G.; Garbit, F.; Grandclement, E.; Vignal, J.; Gilly, F.N. Intraperitoneal chemo-hyperthermia with mitomycin C for gastric cancer patients with peritoneal carcinomatosis. *Anticancer Res* **1999**, *19*, 1375–1382.
125. Fujimoto, S.; Takahashi, M.; Mutou, T.; Kobayashi, K.; Toyosawa, T. Successful intraperitoneal hyperthermic chemoperfusion for the prevention of postoperative peritoneal recurrence in patients with advanced gastric carcinoma. *Cancer* **1999**, *85*, 529–534.
126. Hamazoe, R.; Maeta, M.; Kaibara, N. Intraperitoneal thermochemotherapy for prevention of peritoneal recurrence of gastric cancer. Final results of a randomized controlled study. *Cancer* **1994**, *73*, 2048–2052.
127. Fujimoto, S.; Shrestha, R.D.; Kokubun, M.; Kobayashi, K.; Kiuchi, S.; Konno, C.; Ohta, M.; Takahashi, M.; Kitsukawa, Y.; Mizutani, M., et al. Positive results of combined therapy of surgery and intraperitoneal hyperthermic perfusion for far-advanced gastric cancer. *Ann Surg* **1990**, *212*, 592–596.
128. Koga, S.; Hamazoe, R.; Maeta, M.; Shimizu, N.; Murakami, A.; Wakatsuki, T. Prophylactic therapy for peritoneal recurrence of gastric cancer by continuous hyperthermic peritoneal perfusion with mitomycin C. *Cancer* **1988**, *61*, 232–237.
129. Li, Y.C.; Khashab, T.; Terhune, J.; Eckert, R.L.; Hanna, N.; Burke, A.; Richard Alexander, H. Preoperative Thrombocytosis Predicts Shortened Survival in Patients with Malignant Peritoneal Mesothelioma Undergoing Operative Cytoreduction and Hyperthermic Intraperitoneal Chemotherapy. *Ann Surg Oncol* **2017**, *24*, 2259–2265, doi:10.1245/s10434-017-5834-2.
130. Kepenekian, V.; Elias, D.; Passot, G.; Mery, E.; Goere, D.; Delroeux, D.; Quenet, F.; Ferron, G.; Pezet, D.; Guilloit, J.M., et al. Diffuse malignant peritoneal mesothelioma: Evaluation of systemic chemotherapy with comprehensive treatment through the RENAPE Database: Multi-Institutional Retrospective Study. *Eur J Cancer* **2016**, *65*, 69–79, doi:10.1016/j.ejca.2016.06.002.
131. Huang, Y.; Alzahrani, N.A.; Liauw, W.; Morris, D.L. Repeat cytoreductive surgery and hyperthermic intraperitoneal chemotherapy for recurrent diffuse malignant peritoneal mesothelioma. *Eur J Surg Oncol* **2015**, *41*, 1373–1378, doi:10.1016/j.ejso.2015.07.004.
132. Robella, M.; Vaira, M.; Mellano, A.; Marsanic, P.; Cinquegrana, A.; Borsano, A.; Barbera, M.; Caneparo, A.; Siatis, D.; Sottile, A., et al. Treatment of diffuse malignant peritoneal mesothelioma (DMPM) by cytoreductive surgery and HIPEC. *Minerva Chir* **2014**, *69*, 9–15.
133. Passot, G.; Bakrin, N.; Isaac, S.; Decullier, E.; Gilly, F.N.; Glehen, O.; Cotte, E. Postoperative outcomes of laparoscopic vs open cytoreductive surgery plus hyperthermic intraperitoneal chemotherapy for treatment of peritoneal surface malignancies. *Eur J Surg Oncol* **2014**, *40*, 957–962, doi:10.1016/j.ejso.2013.10.002.
134. Hommell-Fontaine, J.; Isaac, S.; Passot, G.; Decullier, E.; Traverse-Glehen, A.; Cotte, E.; You, B.; Mohamed, F.; Gilly, F.N.; Glehen, O., et al. Malignant peritoneal mesothelioma treated by cytoreductive surgery and

hyperthermic intraperitoneal chemotherapy: is GLUT1 expression a major prognostic factor? A preliminary study. *Ann Surg Oncol* **2013**, *20*, 3892–3898, doi:10.1245/s10434-013-3077-4.
135. Deraco, M.; Baratti, D.; Hutanu, I.; Bertuli, R.; Kusamura, S. The role of perioperative systemic chemotherapy in diffuse malignant peritoneal mesothelioma patients treated with cytoreductive surgery and hyperthermic intraperitoneal chemotherapy. *Ann Surg Oncol* **2013**, *20*, 1093–1100, doi:10.1245/s10434-012-2845-x.
136. Chua, T.C.; Liauw, W.; Saxena, A.; Al-Mohaimeed, K.; Fransi, S.; Zhao, J.; Morris, D.L. Evolution of locoregional treatment for peritoneal carcinomatosis: single-center experience of 308 procedures of cytoreductive surgery and perioperative intraperitoneal chemotherapy. *Am J Surg* **2011**, *201*, 149–156, doi:10.1016/j.amjsurg.2010.02.012.
137. Blackham, A.U.; Shen, P.; Stewart, J.H.; Russell, G.B.; Levine, E.A. Cytoreductive surgery with intraperitoneal hyperthermic chemotherapy for malignant peritoneal mesothelioma: mitomycin versus cisplatin. *Ann Surg Oncol* **2010**, *17*, 2720–2727, doi:10.1245/s10434-010-1080-6.
138. Baratti, D.; Kusamura, S.; Nonaka, D.; Oliva, G.D.; Laterza, B.; Deraco, M. Multicystic and well-differentiated papillary peritoneal mesothelioma treated by surgical cytoreduction and hyperthermic intra-peritoneal chemotherapy (HIPEC). *Ann Surg Oncol* **2007**, *14*, 2790–2797, doi:10.1245/s10434-007-9475-8.
139. Baratti, D.; Kusamura, S.; Cabras, A.D.; Dileo, P.; Laterza, B.; Deraco, M. Diffuse malignant peritoneal mesothelioma: Failure analysis following cytoreduction and hyperthermic intraperitoneal chemotherapy (HIPEC). *Ann Surg Oncol* **2009**, *16*, 463–472, doi:10.1245/s10434-008-0219-1.
140. Elias, D.; Bedard, V.; Bouzid, T.; Duvillard, P.; Kohneh-Sharhi, N.; Raynard, B.; Goere, D. Malignant peritoneal mesothelioma: treatment with maximal cytoreductive surgery plus intraperitoneal chemotherapy. *Gastroenterol Clin Biol* **2007**, *31*, 784–788.
141. Deraco, M.; Nonaka, D.; Baratti, D.; Casali, P.; Rosai, J.; Younan, R.; Salvatore, A.; Cabras Ad, A.D.; Kusamura, S. Prognostic analysis of clinicopathologic factors in 49 patients with diffuse malignant peritoneal mesothelioma treated with cytoreductive surgery and intraperitoneal hyperthermic perfusion. *Ann Surg Oncol* **2006**, *13*, 229–237, doi:10.1245/ASO.2006.03.045.
142. Deraco, M.; Casali, P.; Inglese, M.G.; Baratti, D.; Pennacchioli, E.; Bertulli, R.; Kusamura, S. Peritoneal mesothelioma treated by induction chemotherapy, cytoreductive surgery, and intraperitoneal hyperthermic perfusion. *J Surg Oncol* **2003**, *83*, 147–153, doi:10.1002/jso.10255.
143. Kozman, M.A.; Fisher, O.M.; Rebolledo, B.J.; Valle, S.J.; Alzahrani, N.; Liauw, W.; Morris, D.L. CA 19-9 to peritoneal carcinomatosis index (PCI) ratio is prognostic in patients with epithelial appendiceal mucinous neoplasms and peritoneal dissemination undergoing cytoreduction surgery and intraperitoneal chemotherapy: A retrospective cohort study. *Eur J Surg Oncol* **2017**, *43*, 2299–2307, doi:10.1016/j.ejso.2017.09.009.
144. Baratti, D.; Kusamura, S.; Milione, M.; Pietrantonio, F.; Caporale, M.; Guaglio, M.; Deraco, M. Pseudomyxoma Peritonei of Extra-Appendiceal Origin: A Comparative Study. *Ann Surg Oncol* **2016**, *23*, 4222–4230, doi:10.1245/s10434-016-5350-9.
145. Sparks, D.S.; Morris, B.; Xu, W.; Fulton, J.; Atkinson, V.; Meade, B.; Lutton, N. Cytoreductive surgery and heated intraperitoneal chemotherapy for peritoneal carcinomatosis secondary to mucinous adenocarcinoma of the appendix. *Int Surg* **2015**, *100*, 21–28, doi:10.9738/INTSURG-D-14-00089.1.
146. Kusamura, S.; Baratti, D.; Hutanu, I.; Gavazzi, C.; Morelli, D.; Iusco, D.R.; Grassi, A.; Bonomi, S.; Virzi, S.; Haeusler, E., et al. The role of baseline inflammatory-based scores and serum tumor markers to risk stratify pseudomyxoma peritonei patients treated with cytoreduction (CRS) and hyperthermic intraperitoneal chemotherapy (HIPEC). *Eur J Surg Oncol* **2015**, *41*, 1097–1105, doi:10.1016/j.ejso.2015.04.005.
147. Taflampas, P.; Dayal, S.; Chandrakumaran, K.; Mohamed, F.; Cecil, T.D.; Moran, B.J. Pre-operative tumour marker status predicts recurrence and survival after complete cytoreduction and hyperthermic intraperitoneal chemotherapy for appendiceal Pseudomyxoma Peritonei: Analysis of 519 patients. *Eur J Surg Oncol* **2014**, *40*, 515–520, doi:10.1016/j.ejso.2013.12.021.
148. Arjona-Sanchez, A.; Munoz-Casares, F.C.; Casado-Adam, A.; Sanchez-Hidalgo, J.M.; Ayllon Teran, M.D.; Orti-Rodriguez, R.; Padial-Aguado, A.C.; Medina-Fernandez, J.; Ortega-Salas, R.; Pulido-Cortijo, G., et al. Outcome of patients with aggressive pseudomyxoma peritonei treated by cytoreductive surgery and intraperitoneal chemotherapy. *World J Surg* **2013**, *37*, 1263–1270, doi:10.1007/s00268-013-2000-2.

149. Elias, D.; Honore, C.; Ciuchendea, R.; Billard, V.; Raynard, B.; Lo Dico, R.; Dromain, C.; Duvillard, P.; Goere, D. Peritoneal pseudomyxoma: results of a systematic policy of complete cytoreductive surgery and hyperthermic intraperitoneal chemotherapy. *Br J Surg* **2008**, *95*, 1164–1171, doi:10.1002/bjs.6235.
150. Baratti, D.; Kusamura, S.; Martinetti, A.; Seregni, E.; Laterza, B.; Oliva, D.G.; Deraco, M. Prognostic value of circulating tumor markers in patients with pseudomyxoma peritonei treated with cytoreductive surgery and hyperthermic intraperitoneal chemotherapy. *Ann Surg Oncol* **2007**, *14*, 2300–2308, doi:10.1245/s10434-007-9393-9.
151. Loungnarath, R.; Causeret, S.; Bossard, N.; Faheez, M.; Sayag-Beaujard, A.C.; Brigand, C.; Gilly, F.; Glehen, O. Cytoreductive surgery with intraperitoneal chemohyperthermia for the treatment of pseudomyxoma peritonei: a prospective study. *Dis Colon Rectum* **2005**, *48*, 1372–1379, doi:10.1007/s10350-005-0045-5.
152. Deraco, M.; Baratti, D.; Inglese, M.G.; Allaria, B.; Andreola, S.; Gavazzi, C.; Kusamura, S. Peritonectomy and intraperitoneal hyperthermic perfusion (IPHP): a strategy that has confirmed its efficacy in patients with pseudomyxoma peritonei. *Ann Surg Oncol* **2004**, *11*, 393–398, doi:10.1245/ASO.2004.07.002.
153. Deraco, M.; De Simone, M.; Rossi, C.R.; Cavaliere, F.; Di Filippo, F.; Vaira, M.; Piatti, P.; Kusamura, S. An Italian Multicentric Phase II study on peritonectomy and intra peritoneal hyperthermic perfusion (IPHP) to treat patients with pseudomyxoma peritonei. *J Exp Clin Cancer Res* **2003**, *22*, 35–39.
154. Deraco, M.; Gronchi, A.; Mazzaferro, V.; Inglese, M.G.; Pennacchioli, E.; Kusamura, S.; Rizzi, M.; Anselmi, R.A., Jr.; Vaglini, M. Feasibility of peritonectomy associated with intraperitoneal hyperthermic perfusion in patients with Pseudomyxoma peritonei. *Tumori* **2002**, *88*, 370–375.
155. Chen, M.Y.; Chiles, C.; Loggie, B.W.; Choplin, R.H.; Perini, M.A.; Fleming, R.A. Thoracic complications in patients undergoing intraperitoneal heated chemotherapy with mitomycin following cytoreductive surgery. *J Surg Oncol* **1997**, *66*, 19–23.

© 2019 by the authors. Submitted for possible open access publication under the terms and conditions of the Creative Commons Attribution (CC BY) license (http://creativecommons.org/licenses/by/4.0/).
